# Food ingredients and supplements: is this the future?

**DOI:** 10.1186/1479-5876-10-227

**Published:** 2012-11-19

**Authors:** Patrizia Riso, Laura Soldati

**Affiliations:** 1 DeFENS – Department of Food, Environmental and Nutritional Sciences – Division of Human Nutrition, Università degli Studi di Milano, Milan, Italy; 2 Department of Health Sciences, Università degli Studi di Milano, Milan, Italy

## 

The concept of *adequate nutrition* has been changing in the last years in light of the increased knowledge about the relationship between diet and health and the new evidence on the protective role of numerous bioactive compounds introduced with specific categories of foods. In particular, the concept of adequate nutrition has been substituted with that of *optimal nutrition*, in other terms, attention has been focused on nutritional needs to optimize physiological functions and promote health, minimizing the risk of development of degenerative diseases. Moreover, it is always more clear that nutritional needs can vary depending on the sub-groups of population considered and also on factors related to individual genetic characteristics, as supported by several nutrigenetic and nutrigenomic studies [1].

Parallel to the development of the concept of optimal nutrition for health promotion, the interest for food supplements has been increasing. Supplementation as a general concept is perceived as an easy tool to cover nutritional needs even if there is still an open debate on the reliability and sustainability of this approach.

It is clear that a balanced and varied diet provides all the nutrients and non-nutrient compounds necessary for health promotion and disease risk reduction when associated to a healthy life-style. Nevertheless, dietary behavior is often inadequate above all in some groups of the population and rates of obesity and chronic diseases are increasing worldwide.

At this regard, it is estimated that non communicable diseases (*i.e.* heart disease, stroke, chronic respiratory diseases, type 2 diabetes, hypertension, gallbladder disease, certain forms of cancer) that are now the leading cause of death worldwide will still increase by 2020. The WHO’s World Health Report (2002) considered raised blood pressure, raised cholesterol, tobacco use, alcohol consumption, and overweight as the main risk factors for non communicable diseases and declared that 80% of all the cases of heart disease, stroke and type 2 diabetes and 40% of cancers could be prevented by reducing such risk factors.

Obesity is certainly a major contributor to the global burden of non communicable diseases and disability and in the last years we can speak about an obesity pandemic. The simultaneous increases in obesity in almost all countries (both developed and undeveloped countries) has been suggested to be driven mainly by changes in the global food system producing more processed, affordable, and effectively marketed foods [2].

But also there is great attention to the diabetes epidemic. Prevalence of diabetes is different depending on the country with major epicenters in America and Asia. However, the problem is also unfolding rapidly in Europe and diabetes has doubled in the past ten years, and it has been reported to affect 4.9% of the Italian population.

Finally, it is important to consider all the problems related to the increase in the aging population with changes in the physiology and nutritional needs typical of this life stage [3].

In this general context, the market of food ingredients and supplements is growing very quickly in part due to the wide interest of the research directed to the identification and selection of new potential ingredients with functional and/or preventive characteristics and in part due to the increasing demand of groups of consumers particularly interested in the dietary approach to health and well-being.

The question is whether we really need food supplements or not. In other words, we should understand whether nutritional needs are different in the new scenario (due to the increase of obesity, diabetes and chronic diseases). To answer to this question, the first thing is to consider actual food availability and dietary patterns to understand if they are adequate to cover the nutritional needs and allow healthy food choices.

The answer should be affirmative if we consider that appropriate dietary models exist (e.g. the Mediterranean model). It has been recently reported that a 2 point increase in adherence to Mediterranean diet reduces by 6% cancer incidence or mortality, 10% cardiovascular incidence or mortality and 13% neurodegenerative diseases [4].

However, despite the great promotion of this dietary model even in Mediterranean countries there is low compliance. Thus appropriate dietary models are not often followed by most of the population*.*

When are ingredients and/or supplements promoted? They are encouraged in condition of overweight and obesity, hypo-nutrition or underweight, marginal deficiency of several micronutrients, monotony of the diet, increased needs in specific physiological condition or for specific activities or environmental condition, aging. They are also encouraged to improve well-being and/or decrease disease risk.

The enhanced foods or formulations should in principle significantly affect well-being and performance, reach the proper target of population, be safe and available, not be equivocal or a substitute of a varied diet. But, first of all, they should be supported by research instead of marketing.

This means that functional effects should be scientifically substantiated as requested by the European regulation on nutrition and health claims. Main criticisms arisen from European Food Safety Authority (EFSA) in the evaluation of documentations received for substantiation of ingredients or new food products are related with the quality of studies carried out above all in human subjects and weaknesses in the designs apart from validity of biomarkers exploited and the interpretation of their relationship with body function and disease risk.

In this context, the availability of food ingredients with proved efficacy could certainly become a tool in a global strategy to increase the accessibility to healthy food choices. However, it should be also mentioned the problem related with food supplement sustainability. It should finally reflects the actual need to increase the intake of specific nutrients and non nutrients compounds and this is a complex evaluation that take into account numerous factors and above all the availability of appropriate science-based evidence. While such evidence is slowly growing for some bioactives, an ultimate analysis of the socio-economic and environmental impact of food supplement development should be thoroughly evaluated.
